# Mechanical Behaviour and Microstructural Analysis of Earthen Materials Reinforced with Intensive Agricultural By-Products and Binders

**DOI:** 10.3390/ma17246118

**Published:** 2024-12-14

**Authors:** Ana Cervilla-Maldonado, Ignacio Valverde-Palacios, Francisco Martín-Villegas, Raquel Fuentes-García

**Affiliations:** Department of Building Construction, Technical Upper School of Architecture, University of Granada, Campo del Principe, E18071 Granada, Spain; anaecervilla@correo.ugr.es (A.C.-M.); fracimartin@correo.ugr.es (F.M.-V.); rfuentes@ugr.es (R.F.-G.)

**Keywords:** agricultural residue, fibre waste, agricultural by-product, earth construction, compacted earth

## Abstract

Modern construction is largely dependent on steel and concrete, with natural materials such as earth being significantly underutilised. Despite its sustainability and accessibility, earth is not being used to its full potential in developed countries. This study explores innovative building materials using Alhambra Formation soil (Granada, Spain) reinforced with difficult-to-recycle agricultural waste: polypropylene fibres contaminated with organic matter and leachates. Fibres were added at a ratio between 0.20 and 0.80% of the soil mass, leachates at a ratio between 4.25 and 8.50%, and lime was incorporated at 2.00% and 4.00% for specimens with higher residue content. Physico-mechanical properties, including uniaxial compressive strength and longitudinal strain, were analysed together with the microstructure. The results showed that polypropylene fibres, in comparison to the use of leachates, improved compressive strength and ductility, reaching a compressive strength of 1.76 MPa with a fibre content of 0.40%. On the other hand, this value is 7.4% lower than the reference sample without additives. The fibre-reinforced samples showed a higher porosity compared to the samples with leachates or without additives. This approach highlights the potential of agricultural waste for the development of sustainable construction materials, offering enhancements in the strength and ductility of reinforced soils.

## 1. Introduction

The selection of appropriate construction materials depends on availability, material knowledge, and experience with its workability. Even when these conditions are met, societal acceptance significantly influences material choice. This is particularly relevant for earthen materials, which are highly abundant, energy-efficient to extract, process, and reuse, yet often associated with simpler construction and perceived as less mechanically robust, leading to their underuse and limited awareness of their construction potential [1,2,3].

Historically, earth has been widely used in construction due to its accessibility and versatility, evidenced by numerous preserved rural, civil, and monumental buildings [4,5,6]. Research on earthen materials has increased recently, underscoring their economic and environmental benefits and their role in heritage preservation [7,8,9]. Earthen architecture often depends on regional expertise passed down orally, necessitating modern research and documentation to refine traditional techniques.

Rammed earth is a construction system that has been used for millennia in many regions of the world. Major centres of rammed earth construction include North Africa, Australia, regions of North and South America, China and Europe, including France, Germany, and Spain [10]. This construction system consists of ramming the raw earth in layers inside a wooden formwork, in which the cohesive properties of the clays are complemented by mechanical compression. The use of rammed earth building materials has been the subject of renewed interest as a sustainable construction technique, due to the positive environmental impact and potential energy efficiency that they offer [11,12]. More recent research has concentrated on enhancing the durability and structural integrity of rammed earth, as well as investigating reinforcement techniques [11,13]. Moevus et al. [14] focused their research on the hygrothermal and mechanical properties of compressed earth blocks, adobe, and cob.

Furthermore, the moisture content influences the correct compaction of the soil; therefore, the optimum moisture content is essential to activate the binding capacity of the clays [15,16]. This value ranges between 8% and 9% in the case soil from the Alhambra Formation [17]. The Alhambra Formation is a geological formation SE of Spain composed of gravels and sand in a matrix of reddish clays and silts. In the development of this construction system, the choice of the type of soil, the degree of humidity, as well as the use of binders to guarantee its durability are fundamental. Previous studies give as a reference an optimum moisture content of 8.5% of the weight of the soil in the case of Alhambra Formation soil [17,18] according to the European Standard UNE 103501:1994 [19].

In this area, materials reinforced with polypropylene fibres have advantages such as limited cracking and increased ductility [20]. In addition, the use of these by-products can have advantages in replacing other synthetic fibres such as steel or glass fibres [21,22]. Recent studies have also focused on improving the properties of earth-based construction materials through fibre reinforcement techniques. Extensive research, as exemplified by the studies of Shantanu et al. [23] and Abdelhakim et al. [24], analysed the physical, thermal, mechanical, and durability properties of using natural fibres and polypropylene to enhance the physical–mechanical performance of compacted earth blocks. Additionally, research has demonstrated that the incorporation of fibres can enhance the compressive, flexural, and tensile strength of a material, improve its ductility, and reduce the occurrence of cracks forming [25,26]. In contrast, the research conducted by Bailly et al. [27] focused on the utilisation of natural fibres and biological binders as reinforcement for compacted soil blocks.

One of the principal environmental advantages of this study is the use of a residue from intensive agriculture that is difficult to recycle, because polypropylene is contaminated with organic matter [28,29,30,31]. On the other hand, polypropylene fibres do not have the disadvantages of vegetable fibres, such as degradation associated with fibre weakening in alkaline media, volume variation due to high water absorption, as well as fungal attack [32,33,34]. Regarding the use of leachates as an additive for compacted soil, it should be noted that in recent years, more sustainable alternatives to traditional binders have been developed, focusing on those whose production involves less energy consumption. In this line and in the field of soil stabilisation for roads and paths, enzymatic stabilisers have gained popularity due to their effectiveness in stabilisation and their ease of application [35,36].

The aim of this research is to study the potential and performance of polypropylene fibres and leachates from recycling plants as reinforcement additives for Alhambra Formation soil. These by-products are massively generated in regions with intensive agriculture greenhouses. It is worth noting that the extent of intensive agriculture under plastic in the last two decades has been increasing: the total global area grew from 0.7 million to 3.7 million hectares. Globally, China is the country with the largest area of intensive greenhouse agriculture, while in Europe, the countries with the largest greenhouse areas are Spain, Italy, and France [37,38,39]. This type of intensive agriculture causes a significant residue generation problem in regions with this economic activity. In the region of Almeria (Spain), for instance, 4813 tonnes of polypropylene are produced per year across 35,000 ha of intensive agriculture [30].

In this research, polypropylene was tested at 0.20% to 0.80% of soil weight, and leachates at 4.25% to 8.50%. Mechanical and microstructural evaluations were conducted to determine reinforced soil properties. The novelty of this study aims to demonstrate the feasibility of using residues, such as polypropylene and leachates, as reinforcement in compacted earth constructions. This involves the recycling and reuse of by-products from intensive agriculture in regions with this economic activity, which contributes to the circular economy and to the development of knowledge of sustainable construction techniques.

## 2. Materials and Methods

### 2.1. Materials

#### 2.1.1. Edaphic Soil from Alhambra Formation Soil

**Figure 1:**
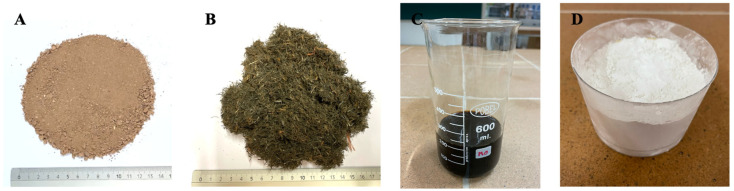
(**A**) Alhambra Formation soil sample. (**B**) Chopped polypropylene fibres. (**C**) Intensive agriculture leachate and (**D**) hydrated lime sample CL 90S.

The base material in this research is soil from the Alhambra Formation (Figure 1A), specifically the B horizon of the edaphic soil mixed with the top of the C horizon. These horizons are rich in quartz, dolomite, and calcite, while the proportion of feldspars, clay minerals, and phyllosilicates is low [40].

The extraction of the soil samples was carried out at the site in the northwest of Granada (Spain), located in El Fargue. For the extraction of the samples, the most superficial layer containing organic remains, known as the A horizon, was removed. In this way, the B horizon could be extracted with almost no organic contamination, thus speeding up the process of cleaning the base material in the laboratory.

The granulometry of the extracted samples is characterised by having an average value of 17.20% in fines (sizes smaller than 0.088 mm sieve), the percentage of sand is 77.90%, and the average percentage of gravel is 4.90% (sizes larger than 5 mm sieve). Specific control tests were carried out to obtain a more detailed characterisation of the Alhambra Formation soil. Once it was confirmed that these results were in accordance with the outcomes of the reference studies [40], it was determined that no further testing was required.

#### 2.1.2. Agricultural By-Products

##### Polypropylene Contaminated with Organic Matter (PP)

**Figure 2:**
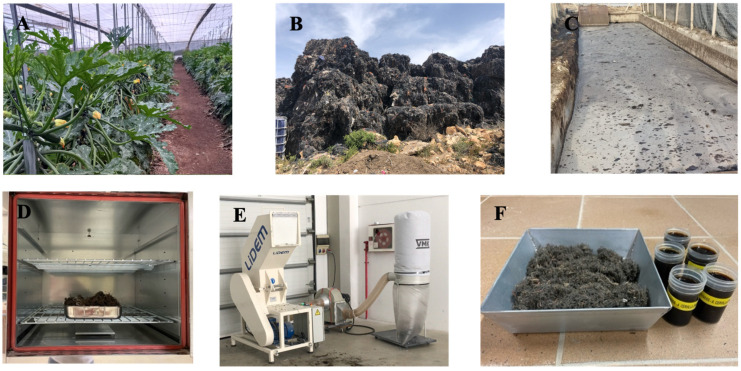
(**A**) Intensive cultivation using polypropylene twines. (**B**) Accumulation of the polypropylene residue at the recycling plant. (**C**) Accumulation of leachate residue at the recycling plant. (**D**) Polypropylene residue oven drying. (**E**) Polypropylene industrial chopping and (**F**) preparation of the residue dosages, polypropylene, and leachate for the compacted earth cylinders.

The polypropylene contaminated with organic matter (Figure 1B and Figure 2B) was provided by the company responsible for the recycling plant for residues from intensive agriculture and is considered a fibre that is difficult to recycle. This is due to the fact that the polypropylene is mostly packed and mixed with organic residues from intensive cultivation (Figure 2A) resulting from the fermentation of organic matter, as well as with metal pieces. The metal parts with a maximum length of 40 mm are used to join the polypropylene raffia on the sides of the horticultural plants and are intended to provide stability to the crops.

Based on previous studies [41,42], it is hypothesised that Alhambra Formation soil reinforced with the polypropylene fibres will show improvements in the mechanical properties of the material, as well as increased ductility.

##### Leachate from Fruit and Vegetable Residues from Intensive Farming (LEA)

The organic leachates used (Figure 1C and Figure 2C) were also provided by the residue recycling plant. The production of the leachate is the result of the fermentation process of organic matter from plant, fruit, and vegetable residues. Therefore, it is hypothesised that leachates are comparable to the enzymes that have been used in previous research for soil stabilisation [43]. They have been applied to improve compaction and to protect soils against weathering and deformation by catalysing a binding action on the plastic particles of cohesive soils [44].

The horizon or layer of the Alhambra Formation soil selected in this study is a cohesive soil with high amounts of clays. Therefore, the material to be developed based on soil reinforced with organic leachates should present improvements in mechanical properties.

##### Hydrated Lime CL 90 S

For this research, hydrated lime CL 90 S powder, produced by the company Ancasa, was used (Figure 1D). This is an aerial lime that consists mainly of CaO (quicklime) and/or Ca(OH)_2_ (calcium hydroxide) without hydraulic or pozzolanic additions [17].

### 2.2. Methods

#### 2.2.1. Treatment of the Soil, Polypropylene and Leachate By-Products for the Production of the Compacted Earth Specimens

Once the previous treatment of cleaning the extracted soil, elimination of organic matter, sieving, and oven drying are carried out (Figure 2D), the polypropylene is industrially chopped (Figure 2E), the leachates are filtered, and the dosages are prepared (Figure 2F). The granulometry of both the soil and the polypropylene fibres is equal to or less than 10mm. The final objective is that the granulometry of the soil and fibres is compatible with the technique for incorporating this material into the system of Projected Earth^®^.

Cylindrical, compacted earth specimens (CESs) were made with dimensions of 100 × 120 mm and a moisture content of 8.5%. The moisture content was established in previous research for soils of the same granulometry and nature [17].

**Figure 3:**
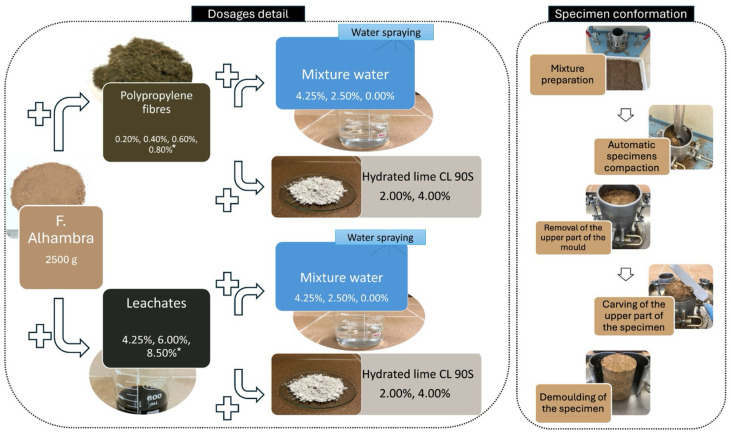
Flowchart with dosages of the compacted earth specimens (CESs) reinforced with polypropylene and leachates, and detail of the specimen conformation. The dosages are calculated in relation to the weight of the soil. * Percentages added with hydrated lime CL 90 S.

The specimens were compacted according to standard UNE 103500:1994 [19]. All the specimens consisted of a base of 2500 g of Alhambra Formation (AF) soil to which different dosages of polypropylene were added (0.20%, 0.40%, 0.60%, and 0.80% of the weight of the soil), as shown in Figure 3 and Table 1. In the case of the leachate-reinforced specimens, the dosage percentages were 4.25%, 6.00%, and 8.50% of soil weight (Figure 3 and Table 1). For this study, reference specimens without additives, consisting only of soil and water, were also prepared.

In addition, after an initial compressive strength analysis, the specimens with the highest by-product dosage of each type were selected and added with 2.00% and 4.00% of hydrated lime CL 90 S. This addition was calculated from the weight of the soil, as shown in Table 2.

Finally, the specimens were dried in the laboratory at room temperature and humidity until they reached a constant weight. The laboratory conditions are defined by a temperature between 20 °C and 24 °C and a humidity between 30% and 40%.

#### 2.2.2. Accelerated Carbonation Process

**Figure 4:**
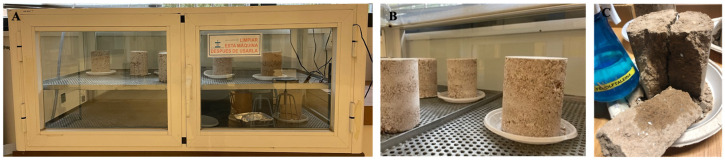
(**A**) Detail of the exterior and (**B**) detail of the interior of the chamber for the accelerated carbonation process. (**C**) Phenolphthalein test on hydrated lime CL 90S-added specimens.

Once the specimens were compacted, the specimens whose dosage contains lime (Table 2) were placed in an airtight chamber connected to a carbon dioxide cylinder (CO_2_). This procedure was carried out in accordance with the standard UNE 83993-2:2013 [45]. In this chamber, temperature conditions of approximately 20 °C and a CO_2_ percentage between 3% and 5% are established, and the specimens are maintained for seven days (Figure 4A,B).

Subsequently, the weights of the specimens were recorded, and the specimens were dried in the laboratory at room temperature and humidity until they reached a constant weight. As all specimens were subjected to the same drying conditions in the same space, the laboratory environment was maintained at consistent temperature and humidity levels. These conditions were defined in the previous section, “Section 2.2.1 Treatment of the soil, polypropylene and leachate by-products for the production of the compacted earth specimens”.

#### 2.2.3. Measurement of Ultrasonic Wave Velocity

After drying the soil specimens, the velocity of the wave through the test specimen is determined according to the UNE-EN 12504-4 standard [46]. This is the velocity reached by the wave to traverse the entire specimen.

The velocity of the wave through the specimen is given by Equation (1), where the distance between sensors (*d*) corresponds to the length of the specimen (120 mm), and the time (*t*) of transmission of the wave between sensors is obtained from the measurement with the Ultrasonic Pulse Analyzer from the Controls company. The calculation of the longitudinal propagation velocity (*v*) of longitudinal wave propagation is expressed in mm/s.

(1) $$v=\frac{d}{t}$$

#### 2.2.4. Unconfined Compression Test

Subsequently, the compressive strength of the specimens was evaluated in accordance with the UNE-EN ISO 17892-7:2019 standard [47]. The objective is to determine the limit load value that the specimens are capable of withstanding and to analyse the deformation of the specimens. The force is applied through the plates of the multi-test press from the company Controls S.A., which has a maximum capacity of 10 T. This test consists of exerting a force at a constant speed of 1 mm/min until the specimen breaks.

**Figure 5:**
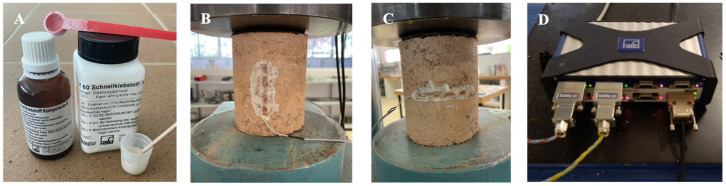
(**A**) Detail of the strain gauges bonding. (**B**) Strain gauge positioned longitudinally to the load. (**C**) Strain gauge positioned transverse to the load and (**D**) QuantumX data acquisition equipment from HBM.

Furthermore, the specimens were instrumented with strain gauges (Figure 5A–C) connected to HBM’s QuantumX data acquisition equipment (Figure 5D) with directions parallel and perpendicular to the axial load, with the objective of obtaining the Poisson’s coefficient (*v*). The Poisson’s coefficient was obtained from the recorded values on the gauges using Equation (2). In these equations, the parameters x and y represent the intervals used to calculate both the longitudinal and transverse deformation (ɛ) until reaching the maximum value before failure.

In order to obtain the Poisson’s coefficient (*ν*), it is necessary to calculate the ratio between the longitudinal and transverse deformation in accordance with Equation (2). This entails determining the reduction in the length of the specimen and the widening in the transverse direction that occurs when the force is applied.

(2) $$\nu =\frac{ɛTx\%-ɛTy\%}{-ɛLx\%-ɛLy\%}$$

#### 2.2.5. Determination of Resistance to Carbonation of the CESs

Once the uniaxial compression breaking process of the specimens is completed, the depth to which the specimens carbonated can be determined by means of the phenolphthalein test, in accordance with the UNE EN 13295:2005 standard [48].

For this purpose, a solution of 1% phenolphthalein in ethanol is applied to the interior of the specimens via spraying. The solution produces a chemical reaction that results in the areas that have not undergone adequate carbonation exhibiting a pink hue, as illustrated in Figure 4C. This method allows for the detection of the depth of carbonation and the assessment of the efficacy of the carbonation process. In other words, the areas of the specimen with a pH higher than 9 (rich in lime) undergo a reddish-pink colour change, while the areas with a pH lower than 9 retain their original colour.

This test is a consequence of the carbonation reaction, whereby calcium hydroxide reacts with carbon dioxide present in the atmosphere to form calcium carbonate and water (Equation (3)).

(3) $$Ca\left(OH\right)2+CO2\to CaCO3+H2O$$

In the absence of carbonation, the deeper the specimen is sprayed with the phenolphthalein solution, the pinker it will become. This phenomenon was observed and documented in order to facilitate the acceleration of the carbonation process. The accelerated carbonation chamber was employed for this purpose, as detailed in chapter ‘Section 2.2.2. Determination of resistance to carbonation of the CESs’.

#### 2.2.6. Distribution and Shape of Cracks in the Specimens After Failure

Following the completion of the unconfined compression test (destructive test), an investigation was conducted into the behaviour of the specimen in response to the applied force. The formation of visible cracks and fissures in the specimens of each type was subjected to detailed analysis.

For the purposes of data collection and visual analysis, a calliper, measuring rulers, and a camera were used. The data were then processed using AutoCAD to create a visual representation of the cracks and fissures, allowing for a detailed analysis of their distribution within the specimen, as well as their verticality, length, and thickness.

#### 2.2.7. Microstructural Characterisation of the CESs with the Inverted Plate Microscope

Optical microscopy was employed for the analysis of the compacted soil specimens under investigation, with three subsamples obtained from disparate points within the specimen undergoing analysis. The method employed enabled the analysis of the porosity by visualising the microstructure of the compacted soil and the distribution of its constituents from its internal structure [49].

A Nikon Epiphot 200 inverted-plate microscope with polarised light was used to perform a detailed microscopic analysis of the specimens, with and without lime, that achieved the best mechanical properties. The optical microscope is equipped with a 7 Mp digital camera connected to a computer, where the digitised image was transformed from pixels to micrometres using Clear Vision’s Perfect Image software, version NIS-BR 4.60x. The resulting image was dependent on the objective used. The magnification was 5×, 10×, 20×, 50×, or 100×.

Sample preparation is a critical factor in achieving optimal results from microscopic observations [50]. Subsequently, the compacted soil cylinders were broken, and the resulting broken cones were meticulously extracted from the selected specimens. Then, these were subjected to a 24 h drying process at 40 °C in an oven. Subsequently, the specimens were subjected to a three-hour tempering process in the tempering chamber.

**Figure 6:**
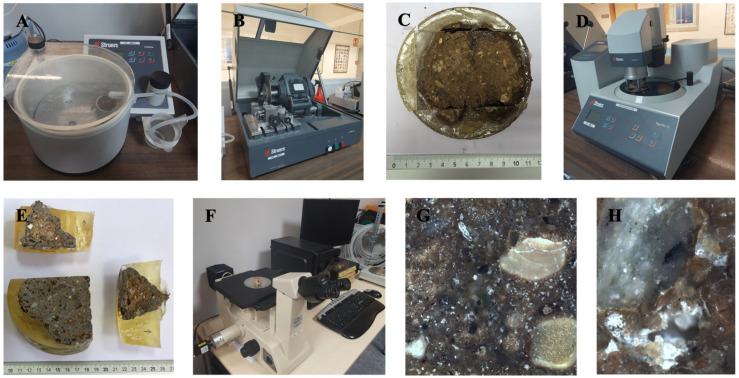
(**A**) Epoxy vacuum impregnation for air bubbles removal and sample fixing. (**B**) Cutting of the samples. (**C**) Fracture cone specimen after the cutting. (**D**) Smoothing and polishing process. (**E**) Samples after the polishing. (**F**) Optical microscope with 7 Mp camera. (**G**) Porosity microscopic analysis of the sample with the 5× objective. (**H**) Cracking and discontinuity analysis of the sample with the 10×/20× objective.

If necessary, the specimens were surface ground to fit into the moulds available for resin treatment, using a metal spatula for grinding. The Struers CitoVac epoxy vacuum machine (4.5 bar) was then used to distribute the low-viscosity Struers Epofix resin, allowing it to penetrate all pores of the cone (Figure 6A). After 24 h of resin curing, the samples were fixed and stable. Subsequently, the specimens were sectioned using the Struers Labotom-3 cutter (Figure 6B,C), thereby ensuring the preservation of the original material structure.

Following the cutting process, the specimens were polished using MD-Piano, DiaPro Allegro, and MD-Dac diamond discs on a Struers TegraPol-11 automatic polishing machine (Figure 6D). In order to achieve an accurate visualisation of the material structure under the microscope, the specimens were polished from higher to lower grit. This process resulted in the elimination of scratches caused by the cut-off wheel. The polishing time was determined individually for each specimen depending on the depth of the scratch marks left by the cutting process (Figure 6E), in order to ensure an accurate visualisation under the microscope (Figure 6F).

In order to obtain the widest possible range of visualisation of the microstructural features of the sample, 30 images of each were captured at 5× resolution (Figure 6G), in accordance with the statistical theory proposed by Bear and Bachmat [51]. This equates to an area of 108,757,777 μm^2^. In the analysed area, higher magnification lenses (10× and 20×) were employed to observe microcracks, smaller diameter pores, and smaller water accumulations in the sample (Figure 6H). In this instance, the porous medium was subjected to an analysis with regard to the void space and the connectivity of the solid matrix. With respect to the two-dimensional plane in which the porosity was analysed, the pores can be classified as predominantly non-connected, with instances of interconnected porosity observed on occasion in areas of high accumulated porosity.

The optical images were binarised from colour to monochrome with the assistance of Clear Vision’s Perfect Image software (version NIS-BR 4.60x), thereby facilitating the interpretation of the observable area and the quantification of the porosity. Furthermore, the distribution of pores in the sample, their shape and size, as well as the total percentage of closed pores, were analysed.

To complete the study of each sample, the images were joined using Adobe Photoshop software 2023 as an auxiliary tool, thereby obtaining a complete view of the analysed area. Additionally, to facilitate the exact quantification of the pores in the sample, AutoCAD 2022 software was used, which allows both the drawing of irregular shapes with greater precision and the total calculation of the porosity area.

## 3. Results and Discussion

### 3.1. Values of Optimum Moisture and Dry Density According to the UNE 103501:1994 Standard [19]

The optimum moisture content and maximum dry density values of the CESs vary between 8.5% and 8.8% and between 18.8 KN/m^3^ and 20.3 KN/m^3^ with the addition of polypropylene, as shown in Table 3. A comparison of these values with those of the reference test tube of AF soil without additives reveals that the addition of polypropylene results in a higher optimum moisture content and a lower maximum dry density. This is due to the fact that polypropylene, which is contaminated with organic matter, requires a greater quantity of water to reach the maximum dry density, due to the absorption capacity of the organic matter. These results are consistent with reference studies using a similar soil type [17].

Furthermore, as the leachate by-product is in liquid form, the optimum moisture value of the reference CES (without additives) was taken for the leachate-reinforced specimens (8.5%).

### 3.2. Unconfined Compression Test According to the Standard UNE-EN ISO 17892-7:2019 [47]

The following graphs illustrate the load capacity supported by each specimen until failure and its resistance over time. In addition, the relationship between compressive strength and longitudinal deformation was also analysed, thanks to the values obtained from the strain gauge placed in the longitudinal direction of the applied load. These results were used to analyse the behaviour and deformation of the specimen as a function of time.

**Figure 7:**
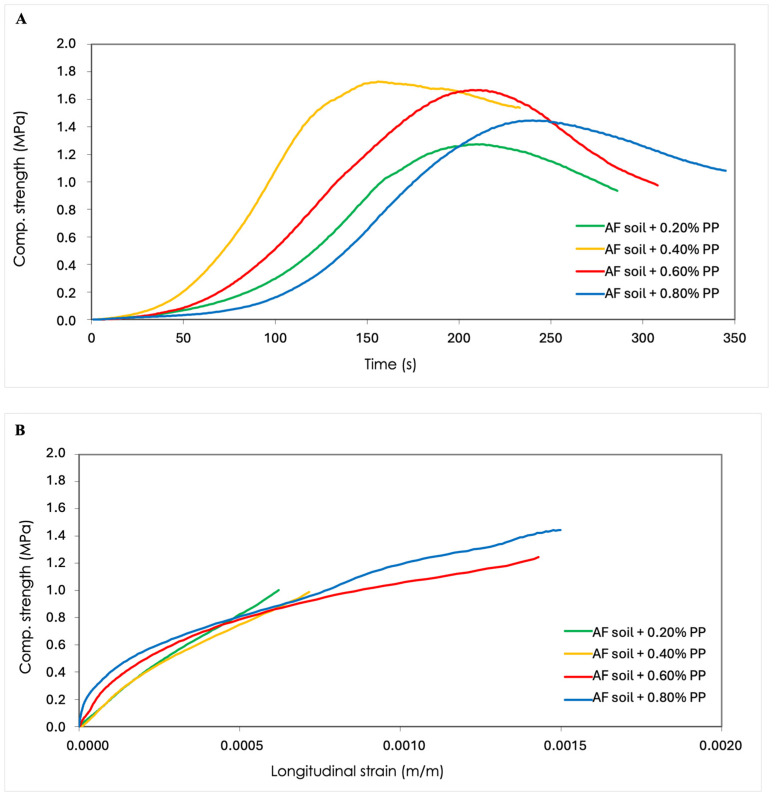
(**A**) Representative compression stress–time curves of the CESs reinforced with polypropylene fibres. (**B**) Stress–strain curves of the CESs reinforced with polypropylene fibres.

As shown in Figure 7A and Table 4, the compressive strengths obtained in the case of the specimens reinforced with polypropylene by-product reached the maximum mean value in the case of the addition of 0.40% residue calculated from the weight of the soil. Moreover, it can be observed that the mean values of compressive strength exhibited a decline with an increase in the percentage of residue above the addition of 0.40% of polypropylene fibres. However, the reduction in compressive strength is not directly proportional to the fibre content, due to the balling effect or inhomogeneous distribution of fibres [52,53].

**Figure 8:**
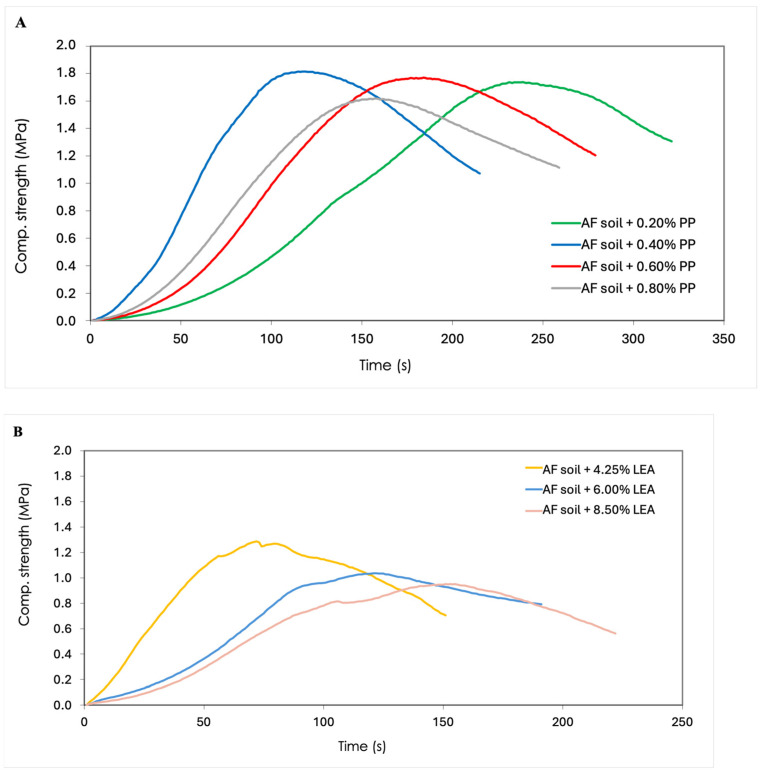
(**A**) Representative compression stress–time curves of CESs with 0.20%, 0.40%, 0.60%, and 0.80% of polypropylene fibres. (**B**) Representative compression stress–time curves of CESs with 4.25%, 6.00%, and 8.50% leachates by-product.

In the case of leachate additions, a decrease in compressive strength was observed as the proportion of residue increased, reaching a maximum average value at the 4.25% leachate dosage. As illustrated in Figure 8B and Table 4, the decline in compressive strength is not directly proportional to the by-product content.

At the initial stage of the deformation process, a linear behaviour is observed until the maximum stress point of each specimen is reached. At this point, the specimen begins to collapse and enters a plastic state, which results in a reduction in compressive strength, as illustrated in Figure 7A,B.

The mean value of the reduction in compressive strength between the 0.20% and 0.80% by-product percentages was 3.1%, while between the 0.40% and 0.80% was 10.2%, as shown in Table 4. The highest mean deviation was observed for the leachate by-product, with a value of 23% between the lowest and highest by-product dosages. This can be attributed to the observed instability in the failure of the leachate specimens, which resulted in a less linear stress–time curve with small jumps, in comparison to the stress–time curves of the polypropylene-reinforced specimens (Figure 8A,B).

On the other hand, the compressive strengths attained in the lime-added specimens (with the highest by-product dosage) were inferior to those observed in specimens without lime, as illustrated in Table 4 and Table 5. The specimens incorporating hydrated lime exhibited mean strength values that were between 20.8% (AF soil specimen + 8.5% LEA + 4.00% CL 90S) and 82.9% (AF soil specimen + 0.80% PP + 4.00% CL 90S) lower than those of the same type without lime.

As shown in Table 4, the highest densities are observed in the specimens with the lowest amount of by-product, with the exception of the specimen added with 0.40% polypropylene. Conversely, the specimens with a greater proportion of by-product exhibited a diminished compressive strength. It is noteworthy that a correlation can be observed between the specimens with higher densities and those with higher compressive strength values.

This can be attributed to the physical properties of polypropylene fibres, which have a lower density than materials with a high fine materials content, such as Alhambra Formation soil. Furthermore, the fibre content of the specimens results in porosity within the matrix, which consequently reduces the density of the composite material. The utilisation of fibre residues has been demonstrated to confer benefits in terms of the lightening and ductility of the composite material, as evidenced by the findings of Falamaki et al. [43].

The negative impact of fibre utilisation can be attributed to their comparatively low stiffness in comparison to sandy materials with a high fine materials content, which ultimately compromises the compressive strength of polypropylene-reinforced CESs. Previous studies have indicated that this is a limiting factor affecting mechanical properties, as well as increasing porosity in the specimen, from the addition of fibres to the matrix [54,55]. The reduction in the interfacial bond between the fibres and the Alhambra Formation soil matrix results in the formation of cracks around the fibres, which in turn accelerates the cracking of the specimen. Furthermore, the inhomogeneous distribution of fibres, or balling effect, also impacts the mechanical strength of the specimen [52,53]. The results displayed in Table 4 demonstrate a consistency in compressive strength and dry density with those observed in previous investigations of compacted soil materials [23,56,57,58,59]. It is important to note that the comparison is based on a range of values and is, therefore, estimated. This is due to the fact that the length of polypropylene used, the nature of the soil, and the additions are not the same as in the present investigation.

On the other hand, the utilisation of leachates was observed to diminish the compressive strength (see Table 4 and Figure 8B). This may be attributed to the penetration of leachate into the soil and alterations in clay structure, as evidenced by reference studies [43]. It is also noteworthy that soil reinforcement with leachate can demonstrate improvements in the short term (7 days) due to the adhesion between the particles. Nevertheless, at longer curing times, the detrimental effects on the structure are more pronounced than the adhesive effects, leading to a reduction in mechanical properties [43]. In our case, the CESs were air-dried for about 30 days to constant weight.

Table 5 illustrates the mechanical properties of specimens that were reinforced with 2.00% and 4.00% of hydrated lime CLH 90S, respectively. In this instance, the dosages with the highest proportion of polypropylene by-product (0.80%) and leachates (8.50%) were selected with the objective of providing a solution to the environmental issue of agricultural residue accumulation. As demonstrated in reference studies, the mechanical properties of the Alhambra Formation soil specimens (without additives) can be enhanced through the addition of hydrated lime [17]. However, given that the specimens in this study were augmented with additional by-products, it can be concluded that the mechanical properties were not enhanced by the addition of hydrated lime CLH 90S to polypropylene and leachate dosages.

Regarding the values of the Poisson’s ratio presented in Table 4, it is worth noting that the ESCs with a greater amount of by-product presented a reduction in this ratio. This coincides with the values obtained in previous studies [60] in which Poisson’s ratio values ranged between 0.37 and 0.15 for the case of soil and cob blocks. In these investigations, as in the results of this study, the highest Poisson’s ratio values were observed in the specimens with the lowest fibre additions. These findings coincide with those observed in specimens exhibiting greater transverse strain than longitudinal strain.

As shown in Table 4, the dynamic modulus of elasticity values was typically lower in the specimens with higher by-product content. This was because the ultrasonic pulse took longer to pass through the specimens with higher by-product content. Conversely, specimens with a residue content of 0.20% polypropylene and 4.25% leachates exhibited higher dynamic modulus of elasticity values. This was because they had less by-product, which constituted less of an obstacle to pulse transmission.

**Figure 9:**
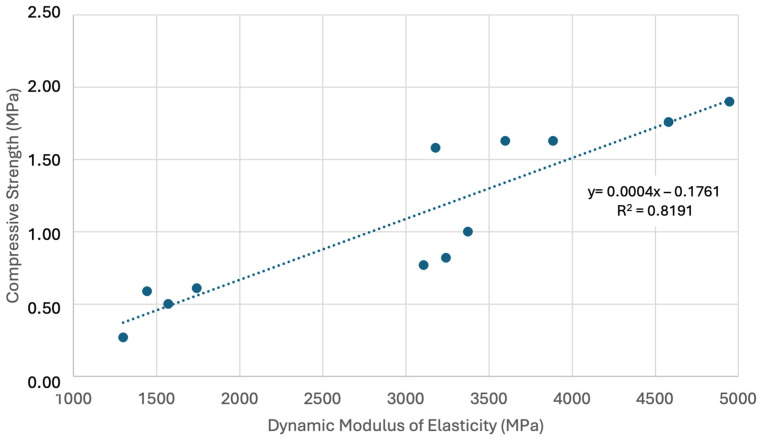
Correlation between the compressive strength and dynamic modulus of elasticity variables.

We analysed the relationship between the residual ratio, Poisson’s ratio, density, and the velocity reached for the ultrasonic pulse to traverse the length of the specimen, as well as the dynamic modulus of elasticity. The results presented in this chapter, in conjunction with those displayed in Table 4 and Table 5, indicate a linear correlation, Compressive Strength (MPa) = 0.0004 * Dynamic Modulus of Elasticity (MPa)—0.1761 (R^2^ = 0.819), between the compressive strength variables and the dynamic modulus of elasticity (Figure 9).

### 3.3. Determination of Resistance to Carbonation According to the Standard UNE-EN 13295:2005 [48]

Once the uniaxial compression breaking process of the specimens was complete, the depth to which the specimens had carbonated was determined by means of the phenolphthalein test. The results of the test demonstrated that all the specimens, which had been treated with hydrated lime CLH 90S and exposed to the accelerated carbonation process, had undergone complete carbonation. Upon the application of a 1% phenolphthalein solution in ethanol to the interior of the specimens, no colour change was observed. This indicated that the carbonation process had been executed correctly because the interior areas of the specimen did not turn pink. The process is shown in Figure 4C.

### 3.4. Crack Analysis on Specimens After Failure

Following the unconfined compression test, polypropylene- and leachate-reinforced specimens were selected for further analysis to investigate the distribution, length, thickness, shape, and verticality of the visible cracks and fissures of each type.

**Figure 10:**
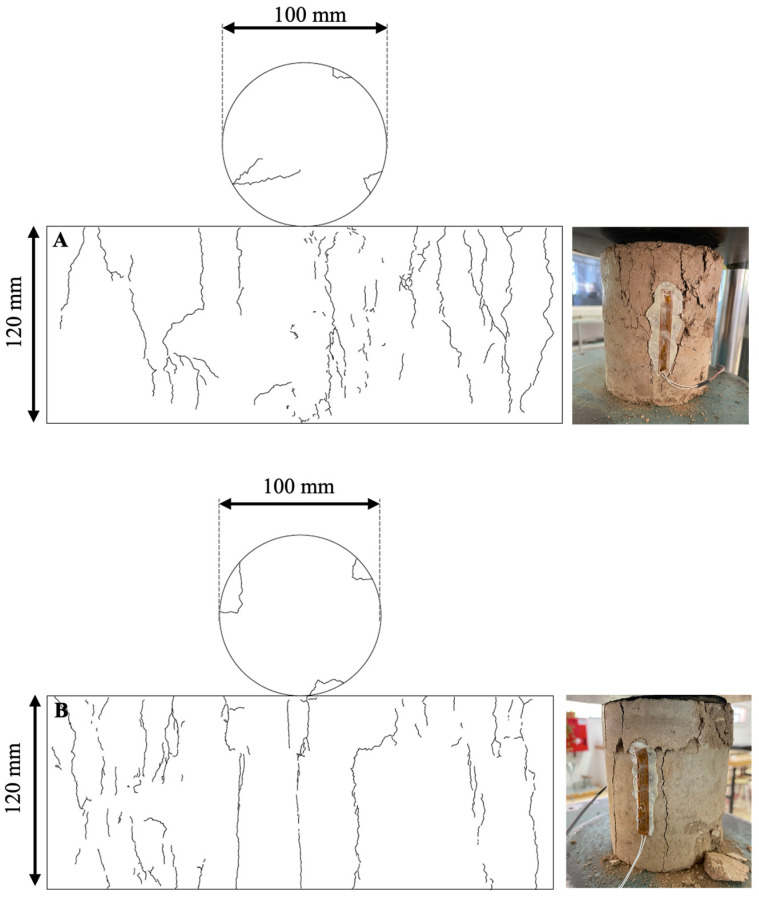
(**A**) Representative diagram of cracks and fissures in polypropylene-reinforced CES. (**B**) Representative diagram of cracks and fissures in leachate-reinforced CES.

As illustrated in Figure 10A,B, the polypropylene-reinforced specimens exhibit a distinct pattern of crack formation and distribution compared to the leachate-reinforced specimens after failure.

The polypropylene-reinforced specimens (Figure 10A) display cracks with less verticality, originating at the top and that do not visibly reach the opposite end of the specimen. The maximum thickness of the visible cracks ranged from 1051 to 2609 μm. These specimens demonstrate minimal material disintegration, occurring only as the specimen reaches its maximum compressive strength capability. This indicates that this specimen type exhibits enhanced stability during the breaking process.

The leachate-reinforced specimens (Figure 10B) display a greater degree of verticality in the cracks, which originate at the top and extend to the opposite end of the specimen. The maximum thickness of the visible cracks ranges from 726 μm to 2197 μm. Additionally, these specimens exhibit material drop-out in regions proximate to the specimen edges where the force is applied. This phenomenon shows that this type of specimen is less stable when reaching its maximum compressive strength capability.

The use of polypropylene fibres in specimen reinforcement has been demonstrated to prevent the formation of weak vertical planes and to exhibit superior ductility behaviour [20,23,25,26,57,58] in comparison to specimens reinforced with leachates.

### 3.5. Microstructural Analysis of Shatter Cones

**Figure 11:**
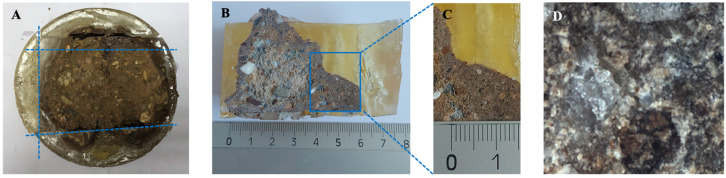
(**A**) Representative breaking cone after resin drying and cutting. (**B**) Sample polishing and selection of one of the scanning areas. (**C**) Macroscopic analysis of the sample and (**D**) microscopic analysis of the sample with the 5× objective.

**Figure 12:**
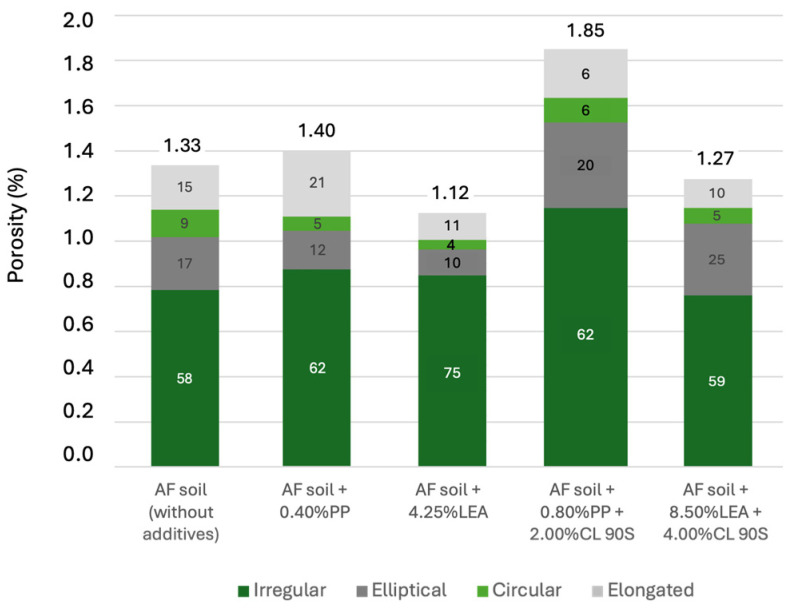
Porosity of the samples (%) and distribution by pore surface according to shape (%).

The porosity and cracking formation of the specimens of each typology (i.e., those reinforced with polypropylene, leachates, polypropylene and lime, and leachate and lime) exhibiting the most favourable mechanical properties were subjected to this analysis and comparison (Figure 11A–C). Subsequently, the results were compared with the microscopic analysis of the reference specimen without additives. This analysis was conducted through direct observation using optical microscopy, as illustrated in Figure 11D. Additionally, Figure 12 presents a quantitative analysis of the porosity, as well as the shape, which was observed to be irregular, elliptical, circular, or elongated.

Figure 11A–C illustrate the process of selection of the scanning zone of one of the breaking cones. In this case, the sample is an AF soil specimen without additives. The porosity results of this sample were compared with those of the breaking cones of each typology. Figure 11B,C show, at the macroscopic level, that the natural aggregates are elongated and oval, and these are dispersed within the soil matrix. Moreover, as evidenced by the reference studies, the natural aggregates present in an AF soil sample exhibit a range of shades, contingent on their intrinsic nature and composition. The dominant whitish natural stones are quartz, dolomite, or calcite, whereas the grey or brown natural stones are phyllosilicates [40].

#### 3.5.1. Breaking Cone Microstructure of CES Reinforced with 0.40% of Polypropylene Fibres

**Figure 13:**
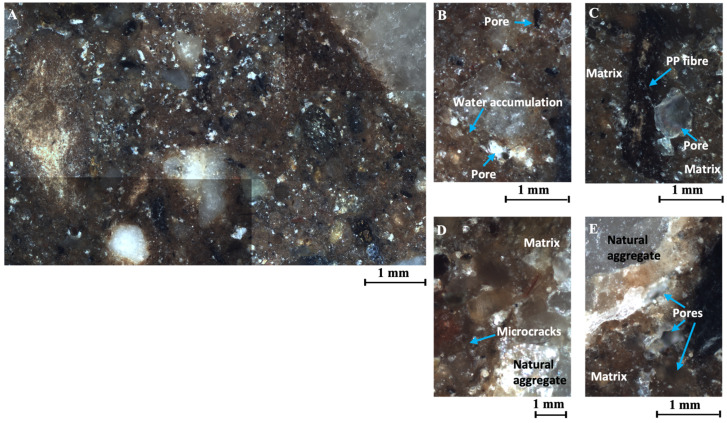
(**A**) Analysis of the specimen of CES reinforced with 0.40% of polypropylene, set of approximately 9 images, (about 56 mm^2^), at 5×. (**B**) Air bubble trapped under natural aggregate larger than 1 mm, at 10×. (**C**) Air bubble trapped under polypropylene fibre, at 10×. (**D**) Microcracks around the natural aggregates, at 20×. (**E**) Porosity next to natural aggregate, at 10×.

The soil specimen reinforced with 0.40% of polypropylene fibres exhibited a higher porosity than the specimen without additives (Figure 13A), with porosity percentages of 1.40% and 1.33%, respectively (Figure 12).

The discontinuity between the polypropylene fibres and the matrix is clearly visible, as evidenced by the presence of pores trapped in the fibres or microporosity that appears around the fibres. This indicates the presence of a potential point of weakness in the matrix–fibre contact zone.

The porosity observed in the sample is randomly distributed throughout the matrix and centred on irregular cracks with an average width of 36 μm. Furthermore, the air pores were produced by air bubbles that were trapped during the mixing process for the conformation of the CESs (Figure 13C). It is noteworthy that the air bubbles are situated in regions exhibiting a change in density, such as the polypropylene fibre–matrix interface or trapped under natural stones larger than 1mm (Figure 13B,E).

On the other hand, the discontinuity between the natural stones and the matrix is minimal and is occasionally marked by the appearance of microcracks bordering the natural aggregate (Figure 13D). The microcracks, measuring approximately 7 μm in thickness, appear during the drying process of the specimen and indicate a point of weakness at the matrix–aggregate interface.

#### 3.5.2. Breaking Cone Microstructure of CES Reinforced with 4.25% of Leachates

**Figure 14:**
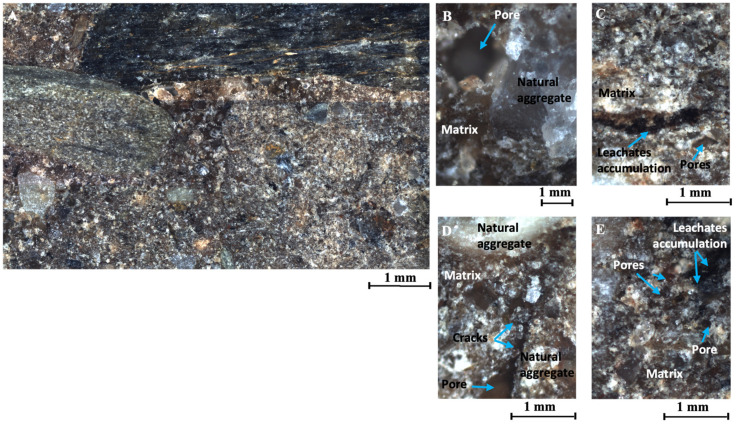
(**A**) Analysis of the specimen of CES reinforced with 4.25% of leachates, set of approximately 9 images, (about 56 mm^2^), at 5×. (**B**) Air bubble trapped under natural aggregate larger than 1 mm, at 20×. (**C**) Leachates accumulation and air bubbles, at 10×. (**D**) Cracks passing through the matrix and bordering the aggregate, at 10×. (**E**) Air bubbles, leachates accumulation and pores, at 10×.

The soil specimen reinforced with 4.25% of leachates shows areas of black or dark grey shading, more visible in the areas of higher leachate concentration (Figure 14C,E). This accentuates the contrast between the matrix and the natural aggregates, improving visualisation under the microscope (Figure 14A). This sample has the lowest porosity of the samples analysed, with a porosity percentage of 1.12% (Figure 12).

The discontinuity between natural stone, matrix, and additives is minimal and mainly characterised by irregular shapes. The irregularly shaped porosity reached 75% of the total porosity analysed (Figure 12) and had an average width of 33 μm. The porosity of the sample is isolated and randomly distributed in the matrix, corresponding to trapped air (Figure 14A). In addition, the formation of air pores during the mixing and compaction process, trapped under the natural aggregates (Figure 14B,D), is also present in this sample.

On the other hand, the appearance of cracks approximately 5.5 μm thick bordering the natural aggregate is due to shrinkage of the matrix during the drying process of the specimen and indicates a point of weakness at the matrix–aggregate interface.

#### 3.5.3. Breaking Cone Microstructure of CES Reinforced with 0.80% of Polypropylene and 2% of Hydrated Lime CL90S

The soil specimen reinforced with 0.80% of polypropylene has the highest porosity of the samples analysed, with a porosity percentage of 1.85% (Figure 12). The porosity of this sample is 32% higher than that of the sample reinforced with 0.40% polypropylene. In general terms, increased porosity also causes a decrease in the ultrasonic wave propagation velocity.

**Figure 15:**
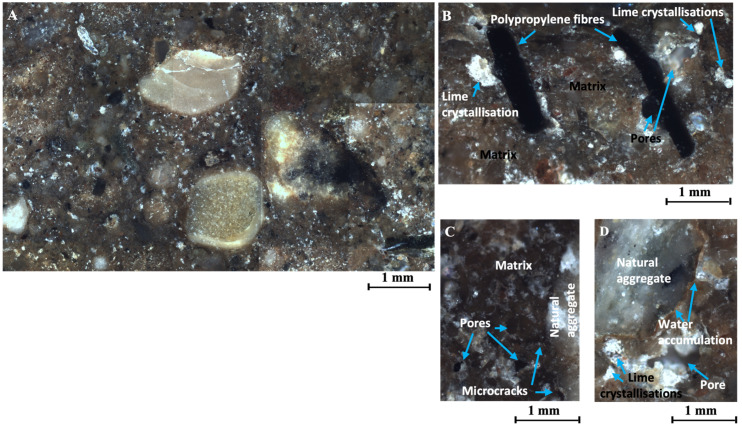
(**A**) Analysis of the specimen of CES reinforced with 0.80% of polypropylene and 2.00% of hydrated lime CL90S, set of approximately 9 images, (about 56 mm^2^), at 5×. (**B**) Air bubbles trapped under polypropylene fibres and lime crystallisations, at 10×. (**C**) Discontinuity due to microcracks bordering the aggregate and pores in the matrix, at 10×. (**D**) Minimum discontinuity at the interface due to water accumulation, at 10×.

The porosity analysed in this sample is randomly distributed throughout the matrix and is centred on irregular cracks with an average width of 29 μm. In this sample, the percentage of porosity with elliptical and circular shapes is higher than in the sample reinforced with 0.40% polypropylene (Figure 12); it is worth noting that this type of porosity usually appears under or next to the polypropylene fibres. This can be seen in Figure 15B where air bubbles are observed next to the polypropylene fibres. The discontinuity between the polypropylene fibres and the matrix is visible by the pores trapped or located around the fibres, indicating a weak point in the matrix–fibre contact zone.

In addition, air pores created by air bubbles entrapped during the mixing process are visible, in this case in the matrix (Figure 15C). The discontinuity between the natural stones and the matrix is generally minimal and is sometimes marked by water accumulation at the periphery of the surface of the larger-diameter aggregates (Figure 15D). This indicates a weak point in the matrix–aggregate contact zone, which is defined as a band of water surrounding the natural aggregate with a maximum thickness of 11 μm.

One of the most representative features of the sample is the presence of bright white crystals corresponding to the crystallisation of the hydrated lime added to the sample. These crystallisations are distributed throughout the sample and have diameters of less than 10 μm in the case of the smallest crystals, and up to 130 μm in the case of the largest crystals (Figure 15A,B,D).

#### 3.5.4. Breaking Cone Microstructure of CES Reinforced with 8.50% of Leachates and 4.00% of Hydrated Lime CL90S

**Figure 16:**
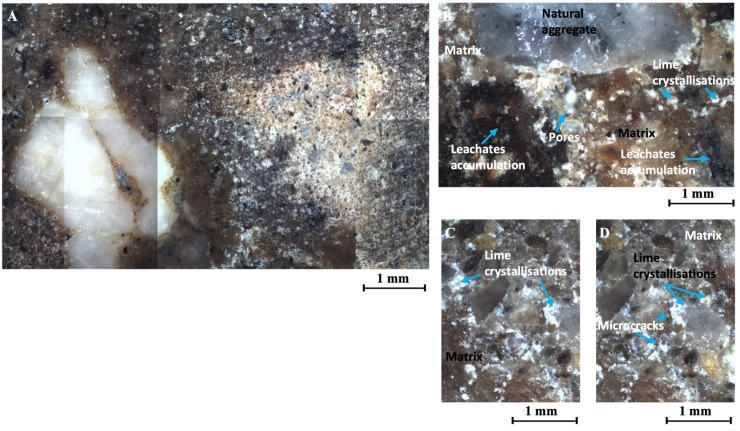
(**A**) Analysis of the specimen of CES reinforced with 8.50% of leachates and 4.00% of hydrated lime CL90S, set of approximately 9 images, (about 56 mm^2^), at 5×. (**B**) Leachate accumulations, air bubbles trapped under natural aggregate and lime crystallisations, at 10×. (**C**) Lime crystallisations bordering the natural aggregates, at 10×. (**D**) Microcracks bordering lime crystallisations next to the aggregates, at 10×.

The soil specimen reinforced with 8.50% of leachates and 4.00% hydrated lime CL 90S has more intense black or dark grey shades than the specimen reinforced with 4.25% of leachates (Figure 16A,B). This accentuates the contrast between the matrix and the natural aggregates.

In addition, the presence of bright white crystals corresponds to the crystallisation of the hydrated lime that was added to the sample. These crystallisations are distributed throughout the sample, concentrating at the edges of the natural aggregates, and have diameters between 6 μm and 107 μm (Figure 16A,C). This sample has a lower porosity than the sample without additives with a porosity of 1.27% (Figure 12).

The discontinuity between the natural stones, the matrix, and the additives is lower than in the other samples analysed. As in the rest of the samples analysed, the porosity with irregular shapes reached a high value; in this case, it was 59%. It should be noted that this sample had the lowest percentage of elongated porosity, only 10% (Figure 12). This type of porosity corresponds to visible cracks in the sample with an average thickness of 32 μm (Figure 16D).

As in the rest of the samples analysed, trapped air bubbles formed during the mixing and compaction of the samples were visualised (Figure 16B). Porosity is randomly distributed throughout the matrix.

## 4. Conclusions

The results of the physical, mechanical, and microstructural tests indicate that the compressive strength of polypropylene- and leachate-reinforced CESs is influenced by variables such as the proportion of by-product used, the particle granulometry of the fibres, and the specimen preparation process. Therefore, the conclusions of this study can be summarised as follows:

- The addition of polypropylene and leachate to the CESs results in a reduction in compressive strength compared to the reference specimens formed with AF soil and water (without additions).
- The addition of 0.40% polypropylene fibres resulted in the highest average compressive strength values compared to other types and percentages of additions. This value is 76.0% higher than the highest value obtained with leachate reinforcement and only 7.4% lower than the average value of the reference unreinforced specimens. Therefore, the addition of polypropylene fibres, regardless of the dosage, improves the residual compressive strength of CES specimens after failure compared to unreinforced specimens. This is due to the improved ductility of the material as a result of the addition of fibres.
- The compressive strength of the leachate-reinforced CESs decreases as the by-product content increases and reaches its maximum value at the lowest dosage, which is 4.25% of leachate.
- The addition of lime to the specimens with the highest by-product content, regardless of the type, did not improve the compressive strength values. On the other hand, the microstructural analysis shows that the specimens reinforced with lime have fewer cracks or fissures, which translates into less elongated porosity.
- The polypropylene-reinforced specimens showed more branched cracks with less verticality compared to the leachate-reinforced specimens. This indicates a ductile behaviour of the material and a less sudden or abrupt failure. On the other hand, the leachate-reinforced specimens show more vertical cracks that occur along the entire length of the specimen.
- The specimens reinforced with polypropylene have a higher porosity than the specimens reinforced with leachates and the specimens without additives. This is due to the pores observed next to the polypropylene fibres. In addition, the fibre clusters lead to higher porosity, which results in less compaction of the material during the specimen forming process. The leachate-reinforced specimens achieved the lowest porosity, even lower than the reference specimen without additives. These positive results in terms of material compaction are not consistent with the compressive strength results obtained with this typology.
- This research shows the influence of the variables (residue type, dosage, and particle size) in achieving the maximum performance of the physical, mechanical, and microstructural properties of CESs. These results are limited to the use of polypropylene fibres and leachates compared to reference specimens without additives, and further research should be carried out with other types of residues. This innovative research employs the use of by-products derived from intensive agriculture, encompassing a comprehensive range of analytical techniques, including the definition of material dosages, mechanical analysis, and microstructural and porosity analysis of the developed materials. This meticulous analysis demonstrates that the use of polypropylene by-products and leachates can be employed in construction projects based on Compacted Earth and Projected Earth^®^ techniques. This represents a significant advantage in the recycling and reuse of by-products derived from intensive agricultural practices. Moreover, this contributes to the circular economy of urban and national economies where this type of economic activity, such as intensive agriculture, is prevalent. The findings of this research will contribute to the further development of earthen materials, providing an affordable and environmentally responsible building material.

## Figures and Tables

**Table 1:** Details of the CESs dosages and descriptions of the experimental tests.

Specimen Name	Residue Type	Mass Soil (g)	Mass Water (g)	By-Product Residue (g)	Experimental Tests
AF soil (without additives)	-	2500	212.5	-	Ultrasonic wave velocity Compressive strength
AF soil + 0.2%PP	Polypropylene fibres	2500	212.5	5.0	Ultrasonic wave velocity Compressive strength
AF soil + 0.4%PP	Polypropylene fibres	2500	212.5	10.0	Ultrasonic wave velocity Compressive strength Microstructural analysis
AF soil + 0.6%PP	Polypropylene fibres	2500	212.5	15.0	Ultrasonic wave velocity Compressive strength
AF soil + 0.8%PP	Polypropylene fibres	2500	212.5	20.0	Ultrasonic wave velocity Compressive strength
AF soil + 4.25%LEA	Intensive agriculture leachates	2500	106.3	106.3	Ultrasonic wave velocity Compressive strength Microstructural analysis
AF soil + 6.00%LEA	Intensive agriculture leachates	2500	62.5	150.0	Ultrasonic wave velocity Compressive strength
AF soil + 8.50%LEA	Intensive agriculture leachates	2500	-	212.5	Ultrasonic wave velocity Compressive strength

**Table 2:** Details of the CESs dosages reinforced with lime and descriptions of the experimental tests.

Specimen Name	Residue Type	Mass Soil (g)	Mass Water (g)	By-Product Residue (g)	Mass CL 90S (g)	Experimental Tests
AF soil + 0.8%PP + 2%CLH 90S	Polypropylene fibres	2500	212.5	20.0	50	Ultrasonic wave velocity Compressive strength Accelerated carbonation chamber Microstructural analysis
AF soil + 0.8%PP + 4%CLH 90S	Polypropylene fibres	2500	212.5	20.0	100	Ultrasonic wave velocity Compressive strength Accelerated carbonation chamber
AF soil + 8.5%LEA + 2%CLH 90S	Intensive agriculture leachates	2500	-	212.5	50	Ultrasonic wave velocity Compressive strength Microstructural analysis Accelerated carbonation chamber
AF soil + 8,5%LEA + 4%CLH 90S	Intensive agriculture leachates	2500	-	212.5	100	Ultrasonic wave velocity Compressive strength Accelerated carbonation chamber Microstructural analysis

**Table 3:** Values of optimum moisture content and maximum dry density.

Specimen Type	Optimum Moisture Content (%)	Maximum Dry Density (KN/m^3^)
AF soil (without additives)	8.5	20.3
AF soil + PP	8.8	18.8

**Table 4:** Chart of results: specimens reinforced with polypropylene and leachates.

Specimen Type	Compressive Strength (MPa)	Poisson	Dry Density (KN/m^3^)	Ultrasonic Pulse Propagation Velocity (m/s·10^−9^)	Dynamic Modulus of Elasticity (MPa)
AF soil (without additives)	1.90 ± 0.29	0.29 ± 0.02	18.6 ± 0.22	1.63 ± 0.06	4946 ± 307
AF soil + Polypropylene					
0.20%	1.63 ± 0.32	0.30 ± 0.03	17.9 ± 0.12	1.45 ± 0.15	3884 ± 796
0.40%	1.76 ± 0.05	0.25 ± 0.02	18.3 ± 0.29	1.58 ± 0.07	4580 ± 456
0.60%	1.63 ± 0.16	0.22 ± 0.01	17.9 ± 0.16	1.41 ± 0.09	3597 ± 464
0.80%	1.58 ± 0.12	0.21 ± 0.02	17.5 ± 0.02	1.35 ± 0.03	3178 ± 167
AF soil + Leachates					
4.25%	1.00 ± 0.26	0.31 ± 0.02	17.9 ± 0.05	1.37 ± 0.06	3373 ± 303
6.00%	0.82 ± 0.25	0.26 ± 0.03	17.8 ± 0.03	1.33 ± 0.14	3241 ± 669
8.50%	0.77 ± 0.16	0.25 ± 0.01	17.8 ± 0.17	1.32 ± 0.06	3106 ± 327

**Table 5:** Chart of results: specimens reinforced with polypropylene, leachates, and lime CL 90S.

Specimen Type	Compressive Strength (MPa)	Poisson	Dry Density (KN/m^3^)	Ultrasonic Pulse Propagation Velocity (m/s·10^−9^)	Dynamic Modulus of Elasticity (MPa)
AF soil + PP + Lime CL 90S					
0.80%PP + 2% CL90S	0.59 ± 0.02	0.25 ± 0.03	16.3 ± 0.06	0.94 ± 0.05	1441 ± 223
0.80%PP + 4% CL90S	0.27 ± 0.07	0.23 ± 0.02	16.2 ± 0.09	0.89 ± 0.02	1299 ± 178
AF soil + LEA + Lime CL 90S					
8.50%LEA + 2% CL90S	0.50 ± 0.06	0.21 ± 0.01	16.4 ± 0.16	0.98 ± 0.06	1569 ± 136
8.50%LEA + 4% CL90S	0.61 ± 0.02	0.18 ± 0.02	16.5 ± 0.11	1.03 ± 0.04	1742 ± 152

## Data Availability

Data will be made available on request.

## Abbreviations

CES: compacted earth specimen; AF: Alhambra Formation; OM: optimum moisture; PP: polypropylene; LEA: leachates.
